# Silver Nanocluster–Based Label-Free Aptasensor for the Turn-On Fluorescent Detection of Ochratoxin A

**DOI:** 10.3390/foods14183271

**Published:** 2025-09-20

**Authors:** Jinyan Nan, Chengbi Cui, Zhijun Guo

**Affiliations:** College of Agriculture, Yanbian University, Yanji 133002, China; nanjinyan0630@163.com (J.N.); chengbicui@ybu.edu.cn (C.C.)

**Keywords:** DNA scaffold, silver nanoclusters, label-free fluorescent aptasensor, ochratoxin A

## Abstract

Despite the substantial human health risks posed by ochratoxin A (OTA), a potent mycotoxin, simple, low-cost methods for its sensitive and selective detection in foods are lacking. To address this gap, we herein developed a label-free OTA aptasensor based on deoxyribonucleic acid (DNA)-scaffolded silver nanoclusters (AgNCs) with an intense red fluorescence. As the DNA template fragment used for AgNC fabrication was derived from the complementary sequence of the OTA aptamer (Apt-OTA), Apt-OTA complexed the AgNCs in the absence of OTA, quenching their fluorescence. OTA inhibited this quenching by strongly binding Apt-OTA and thus precluding its binding to the AgNCs. The OTA aptasensor exhibited a high selectivity and low detection limit (0.38 ng/mL), eliminating the need for expensive reagents, complicated pre-treatments, and advanced equipment, and was successfully used to quantify mycotoxins in food under real-life conditions, thus holding promise for mycotoxin control.

## 1. Introduction

Ochratoxin A (OTA) is a toxic compound generated by various fungal species, including those belonging to the *Aspergillus* and *Penicillium* genera [1,2,3], and is commonly present in foods such as cereals, wine, coffee beans, nuts, milk, eggs, meat, and animal feed [4,5]. This mycotoxin poses considerable risks to human and animal health, having the capacity to damage the kidneys, developing embryos, liver, nervous system, and immune system and increase the risk of cancer [6,7,8]. In 1993, the International Agency for Research on Cancer categorised OTA as a Group 2 B carcinogen, which inspired numerous countries and regions to establish maximum allowable OTA limits for food products [9]. Currently, OTA detection largely relies on high-performance liquid chromatography (HPLC), liquid chromatography-tandem mass spectrometry, and enzyme-linked immunosorbent assays [10,11,12]. Despite their effectiveness, these methods exhibit several drawbacks, e.g., substantial expenses, extended analysis durations, and reliance on skilled operators and specialised equipment, and are therefore poorly suited for field-based detection. Additionally, preparing antibodies against small-molecule targets is complicated and expensive, while immunoassays usually involve labelling processes that further increase the cost—exacerbating the limitations of traditional detection methods for on-site applications. Hence, efficient and sensitive OTA detection methods enabling the issuance of early warnings to ensure food safety are urgently required.

Nanomaterials are widely used in fluorescence assays because of their advantageous optical, physical, and chemical properties and enable the downsizing of detection equipment and development of rapid, precise, efficient, sensitive, and portable pathogen diagnostic tools [13,14]. Such systems can be easily operated by non-specialised personnel and require minimal sample quantities for the on-site measurement of the target parameters. Metal nanoparticles, graphene, carbon nanotubes, nanowires, and other nanomaterials frequently exhibit size-dependent catalytic activity and other advantageous properties, having a substantial application potential and holding promise for analytical tool development [15,16,17]. Metal (e.g., Ag, Au, and Cu) nanoclusters can exhibit antibacterial effects and have been extensively applied in biosensing, bioimaging, drug delivery, and immunostimulation because of their biocompatibility, high stability, tuneable fluorescence, facile synthesis, and other advantages [18,19].

Deoxyribonucleic acid (DNA)-scaffolded silver nanoclusters (AgNCs) spontaneously form through the assembly of silver atoms/ions and DNA templates rich in cytosine [20,21,22] and integrate the readily customisable nature of DNA with the distinctive optical properties of silver. The first DNA-scaffolded metal nanoclusters were synthesised for metal = silver via a random screening method by the Dickson group, featuring blue, green, yellow, or red emission, depending on the sequence [23]. The fluorescence emission wavelengths of DNA-scaffolded AgNCs (DNA-AgNCs) span the visible-to-near-infrared range and are strongly dependent on the sequences and structures of the employed DNA templates [24]. Furthermore, DNA-AgNCs can inherently recognise DNA and aptamers, and single-stranded DNA templates can be directly linked to aptamers to create aptamer-functionalised label-free complementary strand [25]. The fluorescence quenching of DNA-AgNCs upon the hybridisation of the template DNA with complementary microRNAs can be used for the rapid and straightforward detection of the latter [26]. Herein, a strand complementary to the aptamer was designed. Employing DNA-AgNCs (subsequently referred to as simply AgNCs) reduces complementary strand synthesis expenses while boosting design versatility: the NC core’s tunable optical properties (e.g., emission wavelength, quantum yield) and inherent compatibility with DNA-based modifications let the system be flexibly reconfigured for different analytes or detection scenarios, underscoring the NCs’ key role in expanding the sensor’s applicability. Liu et al. designed a DNA-Cu/Ag NC–based method for the highly sensitive and specific fluorescence activation–based detection of kanamycin [27] offering the advantages of low cost, ease of use, and operational simplicity and eliminating the need for chemical modifications, enzymatic processes, organic cosolvents, and complex instrumentation.

While existing AgNC-based sensors frequently depend on enzyme-aided signal amplification or exogenous chemical labelling (e.g., fluorescent dyes, quantum dots) [28,29] to attain adequate sensitivity—introducing operational complexity, elevated costs, and potential matrix interference—our design obviates these drawbacks by capitalising on the intrinsic red fluorescence of DNA-templated AgNCs and their specific hybridization with the Apt-OTA. Furthermore, among the limited aptamer-DNA-AgNCs sensors reported to date, most are predominantly validated for solid grain matrices (e.g., wheat, corn) [30,31]—a critical limitation that creates a significant technical gap in the rapid detection of OTA in liquid food products (e.g., red wine). Specifically, the high alcohol content and polyphenolic compounds in these liquid matrices readily disrupt the structural stability of AgNCs and interfere with the specific binding interaction between aptamers (Apt-OTA) and OTA, ultimately leading to unreliable and inaccurate detection readouts—a longstanding challenge that has rarely been effectively addressed in prior research. To address these unmet challenges and achieve field-based OTA sensing, we herein developed a label-free OTA aptasensor based on AgNCs with an intense red fluorescence, integrating the unique recognition capability of aptamers with the compact size and tuneable fluorescence properties of AgNCs and thus enabling rapid fluorescence-based OTA detection via energy transfer. Given that the employed DNA template was rich in cytosine and exhibited a partial complementarity with the OTA aptamer (Apt-OTA), the fluorescence of AgNCs was substantially quenched upon their hybridisation with Apt-OTA. This approach enabled the highly sensitive and specific visual/quantitative detection of OTA, holding promise for its detection and that of other foodborne toxins.

## 2. Materials and Methods

### 2.1. Materials

Ochratoxin A aptamer (Apt-OTA) (5′-GAT CGG GTG TGG GTG GCG TAA AGG GAG CAT CGG ACA-3′) [32], zearalenone aptamer (Apt-ZEN), aflatoxin B1 aptamer (Apt-AFB1), chloramphenicol aptamer (Apt-CHL), and kanamycin aptamer (Apt-KAN) were synthesised by Shanghai Sangon Biotechnology Co., Ltd. (Shanghai, China). Ochratoxin A (OTA, purity ≥ 98%, USP grade), ochratoxin B (OTB, purity ≥ 97%), aflatoxin B1 (AFB1, purity ≥ 98%), aflatoxin M1 (AFM1, purity ≥ 97%), and N-acetyl-L-phenylalanine (NAP, purity ≥ 99%, analytical grade) were purchased from Sigma-Aldrich (St. Louis, MO, USA). Agarose (electrophoresis grade, purity ≥ 99.0%), 50 × TAE buffer (molecular biology grade, components: Tris base ≥ 99.8%, acetic acid ≥ 99.5%, EDTA ≥ 99.0%), the DNA ladder (50–400 bp, purity ≥ 98% by agarose gel electrophoresis), and the DNA loading buffer (6×, containing bromophenol blue ≥ 98% and xylene cyanol FF ≥ 98%) were purchased from Solarbio Life Science Co., Ltd. (Beijing, China). Analytical-grade AgNO_3_ (purity ≥ 99.5%, trace metals content ≤ 0.001%), NaBH_4_ (purity ≥ 98%, reagent grade), NaH_2_PO_4_ (purity ≥ 99.0%, analytical grade), and Na_2_HPO_4_ (purity ≥ 99.0%, analytical grade) were purchased from Sinopharm Group Chemical Reagent Co., Ltd. (Beijing, China). Aptamer–OTA binding was conducted in a Tris buffer solution composed of 10 mM Tris (purity ≥ 99.8%, reagent grade), 120 mM sodium chloride (NaCl, purity ≥ 99.5%, analytical grade), 20 mM calcium chloride (CaCl_2_, purity ≥ 99.0%, analytical grade), and 5 mM potassium chloride (KCl, purity ≥ 99.5%, analytical grade), with the pH adjusted to 7.4 using 0.1 M HCl (analytical grade, purity ≥ 36.0%) and 0.1 M NaOH (analytical grade, purity ≥ 96.0%)—both purchased from Sinopharm Group Chemical Reagent Co., Ltd. (Beijing, China). Red wine was manufactured by COFCO Great Wall Wine Co., Ltd. (Hebei, China). All oligonucleotides were purified by HPLC, with their sequences listed in Table S1. The remaining chemicals were of analytical purity (purity ≥ 95%) and used as received without further purification.

### 2.2. Instrumentation

Fluorescence spectra were measured with a Cary Eclipse fluorescence spectrophotometer (Varian, Palo Alto, CA, USA). Emission spectra were recorded at 580 and 700 nm upon excitation at 560 nm, with the excitation and emission slit widths set at 5 nm. Ultraviolet–visible (UV–vis) spectra were acquired using a UV-2600i spectrophotometer (Shimadzu, Kyoto, Kyoto Prefecture, Japan). HPLC chromatographic profiles were acquired using an Agilent 1260 HPLC system (Agilent Technologies, Palo Alto, CA, USA) equipped with a fluorescence detector. Transmission electron microscopy (TEM) imaging (JEM-2100Plus, JEOL, Chiyoda-ku, Tokyo, Japan) was performed at an accelerating voltage of 120 kV. All experiments were conducted at room temperature.

### 2.3. AgNC Synthesis

DNA template (15 μL, 100 μM) and AgNO_3_ (5 μL, 3 mM) solutions were combined, and the volume was adjusted to 95 μL using 0.01 M phosphate buffer (pH 7.4). The mixture was vigorously shaken for 10 s, incubated in the dark at room temperature of approximately 25 °C for 20 min, supplemented with a freshly prepared 3 mM NaBH_4_ solution (2.5 μL), and vigorously shaken for 10 s. The reduction reaction was conducted at 4 °C in the dark for 2 h. The final synthetic molar ratio is DNA:Ag^+^:NaBH_4_ = 1:10:5.

### 2.4. Fluorescent Detection of OTA

The OTA standard solution was diluted to the desired concentration with the Tris buffer (pH 7.4). The resulting OTA standard solution (94 μL) was mixed with the Apt-OTA working solution (20 μM, 5.5 μL). The mixture was incubated at room temperature for 30 min, and a 75 μL aliquot was combined with a 10.7 μM AgNC solution (75 μL) in a centrifuge tube and incubated for 30 min at the same temperature. Fluorescence intensity was subsequently measured using a fluorescence spectrophotometer at an excitation wavelength of 560 nm.

For method validation of this protocol (to ensure the reliability of OTA quantification), the OTA standard solution was further diluted with Tris buffer (pH 7.4) to a series of concentrations: 0, 5, 10, 20, 50, 100, 200, 500, 1000, and 2000 ng/mL. Each diluted standard was analysed via the same incubation (with Apt-OTA and subsequent AgNCs) and fluorescence measurement steps described above, and the fluorescence intensity-concentration relationship was used to construct a calibration curve.

### 2.5. Specificity of OTA Detection

To evaluate the specificity of the developed aptasensor for ochratoxin A (OTA) detection, two sets of experiments were performed to verify the specific interaction between AgNCs and Apt-OTA, as well as the specific recognition of OTA by Apt-OTA. All experiments were conducted in triplicate to ensure reproducibility.

#### 2.5.1. Specificity Verification of Aptamer-AgNCs Interaction

Five HPLC-purified aptamers were prepared in Tris buffer (pH 7.4) to a final concentration of 20 μM, including Apt-OTA, Apt-ZEN, Apt-AFB1, Apt-CHL, and Apt-KAN. AgNCs solution (75 μL) was mixed with each aptamer solution (75 μL) at a molar ratio of AgNC:aptamer = 1:0.15. The mixture was incubated at room temperature for 30 min to allow sufficient interaction.

#### 2.5.2. Specificity Verification of OTA Recognition by Apt-OTA

Initially, the OTA standard and four interfering mycotoxins/analogues (OTB, AFM1, NAP, and AFB1) were diluted to 1000 ng/mL with Tris buffer (pH 7.4). Subsequently, each mycotoxin solution (94 μL) was mixed with Apt-OTA working solution (20 μM, 5.5 μL) and incubated at room temperature for 30 min to allow specific binding. Ultimately, the pre-incubated mixture (75 μL) was added to 75 μL of AgNCs solution (10.7 μM) and incubated at room temperature for another 30 min.

### 2.6. Application

The applicability of our sensor to the analysis of real samples was examined by analysing red wine spiked with OTA at 20, 60, 100, 140 and 180 ng/mL. Detailed content has been provided in the Supplementary Materials.

### 2.7. Statistical Analysis

Statistical analyses were performed using OriginPro 2025 software, while Nano Measurer 1.2.5 software was specifically utilized for particle size analysis.

## 3. Results and Discussion

### 3.1. Design Strategy for OTA Detection

**Scheme 1:**
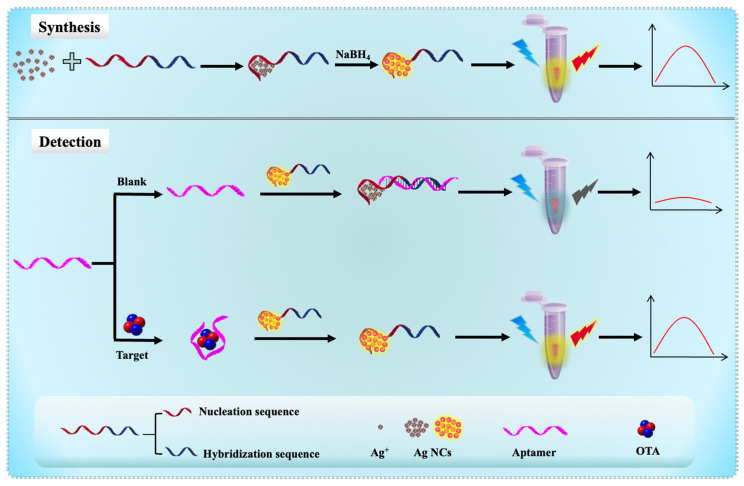
Principle of ochratoxin A (OTA) detection.

The aptasensor design leveraged the fluorescence modulation of the AgNCs by Apt-OTA, with the detection principle illustrated in Scheme 1. The AgNCs were synthesised in situ on DNA bases using a DNA template as a structural framework. When multiple silver ions accumulated on the DNA bases, the local concentration of these ions in the solution increased. Upon reduction with NaBH_4_, these silver ions aggregated to form Ag–Ag bonds, ultimately affording AgNCs with an intense red fluorescence. The template DNA was composed of nucleation (red) and hybridisation (blue) sequences in Scheme 1. Although the nucleation sequence remained unchanged, the fluorescence intensity of AgNCs was notably affected by the base composition of the hybridisation sequence. In the absence of OTA, the AgNCs experienced fluorescence quenching due to hybridisation with Apt-OTA, as the DNA template partially complemented Apt-OTA. Conversely, OTA inhibited this quenching by strongly binding Apt-OTA. Specifically, as a small-molecule mycotoxin, OTA binds to Apt-OTA through three key non-covalent interactions: it first uses its carbonyl, hydroxyl, and amide groups to form hydrogen bonds with Apt-OTA’s nucleobases and sugar-phosphate backbone, which lays the foundation for the specificity of their binding; second, its hydrophobic aromatic rings interact with Apt-OTA’s hydrophobic regions (derived from stacked nucleobases), a process that boosts the stability of the OTA-Apt-OTA complex and ensures OTA binds Apt-OTA more tightly than Apt-OTA binds the DNA template (A1); additionally, driven by the aforementioned hydrogen bonding and hydrophobic interactions, OTA and Apt-OTA’s nucleotide residues draw close, generating weak but cumulative Van der Waals forces that further strengthen their binding—ultimately allowing OTA to competitively bind Apt-OTA (over the DNA template) even at low concentrations. Consequently, the residual fluorescence intensities were correlated with the OTA concentrations.

### 3.2. Selection of Template DNA for AgNCs

**Figure 1:**
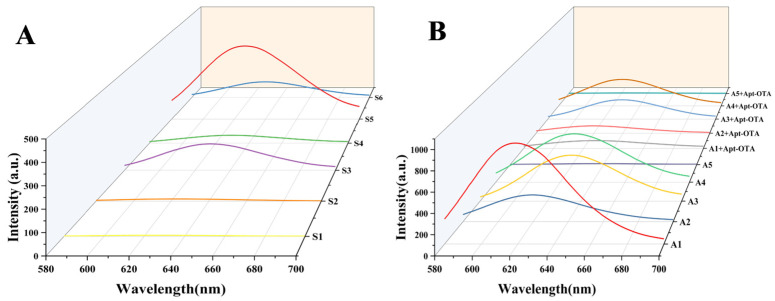
Optimisation of silver nanocluster (AgNC) template sequences. (**A**) Fluorescence spectra of the AgNCs synthesised using S1–S6. (**B**) Fluorescence spectra of the AgNCs synthesised using A1–A5 before and after hybridisation with the OTA aptamer (Apt-OTA).

The brightness of AgNC fluorescence was considerably affected by the base sequence of the template. Thus, the DNA template sequences were assessed and chosen based on their relationship with the fluorescence output of the AgNCs and associated hybridisation sequence. In the design of DNA templates for AgNCs, the nucleation sequence is not arbitrarily constructed. Instead, it is based on three core principles: the interaction rules between DNA and silver ions (Ag^+^), the nucleation and growth requirements of AgNCs, and the structural characteristics of DNA itself. These principles collectively enable the regulation of the nucleation process of AgNCs. In S1 and S2, the 3′ end of the nucleation sequence (5′-CCCCCTTAATCCCCCC-3′) was attached to a sequence composed solely of A and T, and the AgNCs therefore exhibited a negligible fluorescence (Figure 1). In S3–S5, A and T in the aforementioned sequence were randomly substituted with C and G, which substantially increased the fluorescence intensity [33]. The primary purpose of evaluating templates S1–S6 was to verify how the hybridization region’s base composition affects AgNCs fluorescence (confirming cytosine/guanine (C/G) enrichment is essential for intense fluorescence, a foundational step); these templates were “base-composition templates” without targeted complementarity to Apt-OTA—unlike A1–A5 (engineered by ligating C/G-rich fragments from the Apt-OTA complementary strand, Figure S1)—leading to extremely low hybridization efficiency that fails to validate the aptasensor’s core mechanism (“Apt-OTA-template complementarity mediating fluorescence quenching”). In contrast, A1–A5′s designed partial complementarity to Apt-OTA ensures observed quenching (Figure 1) is specific, so excluding S1–S6′s Apt-OTA hybridization data was deliberate: we first established the fluorescence base-composition requirement via S1–S6, then validated the key Apt-OTA-template interaction with A1–A5, avoiding redundancy and enhancing result reliability. Silver ions can effectively bind to DNA bases when AgNO_3_ is mixed with a DNA template, with the binding preference following the order C > G > A > T [34,35]. Consequently, C- and G-rich fragments were selected from the complementary strand of Apt-OTA and ligated to the 3′ end of the nucleation sequence to generate five DNA templates (A1–A5). The complementary binding sites between Apt-OTA and templates A1–A5 are shown in Figure S1. The AgNCs synthesised using A1 exhibited the highest fluorescence intensity (Figure 1). Additionally, Apt-OTA quenched the fluorescence of all AgNCs synthesised using A1–A5 to varying extents. This indicated that the quenching ability may be related to the template sequence and corresponding complementary binding sites. Considering the excellent fluorescence intensity and suppression efficiency observed for A1, it was selected as the optimal template for AgNC synthesis. The sequences of S1–A5 are listed in Table S1.

### 3.3. Optimisation of AgNC Preparation Parameters

Given the compositional variability of the DNA template, the fluorescence properties of the AgNCs may be influenced by the DNA:Ag^+^ concentration ratio. DNA:Ag^+^ concentration ratios of 1:6, 1:12, and 1:17 have been commonly used in AgNC synthesis [36,37]. Consequently, we optimised this ratio and observed its effects on the fluorescence properties of AgNCs (ratios of 1:8, 1:10, 1:12, 1:14, and 1:16 were used).

At a DNA:Ag^+^ concentration ratio of 1:10, the fluorescence intensity reached its optimal value, and this ratio was therefore the most suitable (Figure S2A). When the ratio was set to 1:8, the fluorescence intensity was relatively low, probably because of the insufficient silver ion concentration and, consequently, reduced AgNC formation. When the ratio exceeded 1:10, the fluorescence intensity decreased, particularly at 1:16, in which case almost no emission was observed. This phenomenon was attributed to the excessive aggregation of the AgNCs resulting in the self-quenching of fluorescence and, hence, its notable reduction or complete disappearance. Therefore, a DNA:Ag^+^ ratio of 1:10 was chosen for subsequent experiments. The fluorescence intensity at a DNA:Ag^+^:NaBH_4_ molar ratio of 1:10:5 exceeded that at 1:10:10 by 90.4% (Figure S2B). This enhancement was attributed to the fact that the silver atoms in the AgNC core were stabilised by the surrounding silver ions. Consequently, the complete reduction of Ag+ was not necessary for AgNC formation in the presence of excess NaBH_4_.

At a DNA:Ag^+^:NaBH_4_ ratio of 1:10:5, the fluorescence intensity of the sample synthesised at 4 °C was 24% higher than that of the sample synthesised at 25 °C (Figure S2C). Furthermore, the reaction rate could be effectively controlled at low temperatures to minimise agglomeration and enhance stability. Given that the synthesis temperature of 4 °C was reported to favour the formation of stable AgNCs [38], all subsequent experiments were conducted at 4 °C.

The pH value profoundly influences the physicochemical behaviours of silver nanoclusters by regulating their surface ligand states, size, structure, optical properties, and stability. Most silver nanoclusters exhibit optimal stability under nearly neutral conditions (pH 6–8), where the ligand charge is moderate and the electrostatic repulsion between clusters is balanced, thus preventing aggregation. Appropriate and consistent pH conditions and the ionic environment maintained by the buffer solution play crucial roles in the accurate synthesis of AgNCs. pH influences the protonation of cytosine, which weakens the C–Ag+–C interaction and thus affects the fluorescence intensity of the AgNCs [39,40]. For the AgNCs synthesised in PB buffer at pH 6.6, 7.4, and 8.2, the strongest fluorescence emission occurred at pH 7.4 (Figure S2D). Specifically, the fluorescence intensity at pH 7.4 was approximately 1.2 times higher than that at pH 8.2, and almost no detectable fluorescence was observed at pH 6.6.

The optimal conditions for synthesising AgNCs (A1 templated) were therefore determined to be a PB buffer with a pH of 7.4, DNA:Ag^+^:NaBH_4_ molar ratio of 1:10:5, and an incubation temperature of 4 °C.

### 3.4. Characterisation of AgNCs and AgNCs + Apt-OTA Mixtures

**Figure 2:**
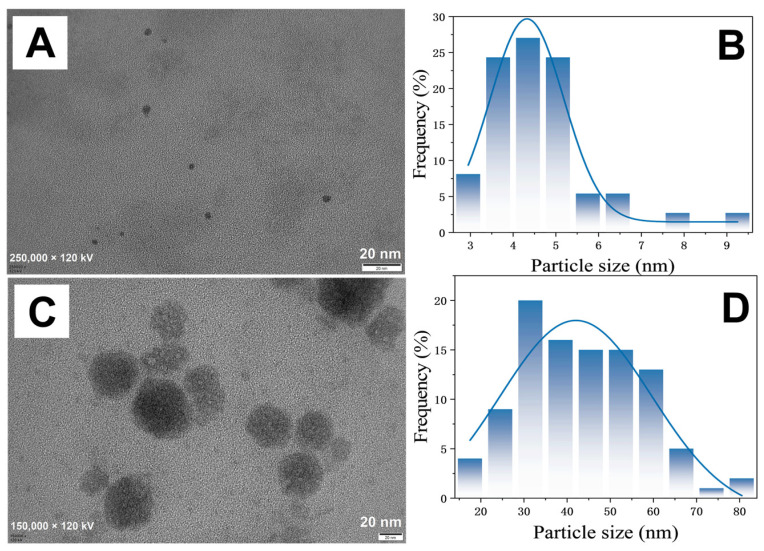
(**A**,**C**) Transmission electron microscopy images and (**B**,**D**) particle size distributions of (**A**,**B**) AgNCs and (**C**,**D**) AgNCs + Apt-OTA.

**Figure 3:**
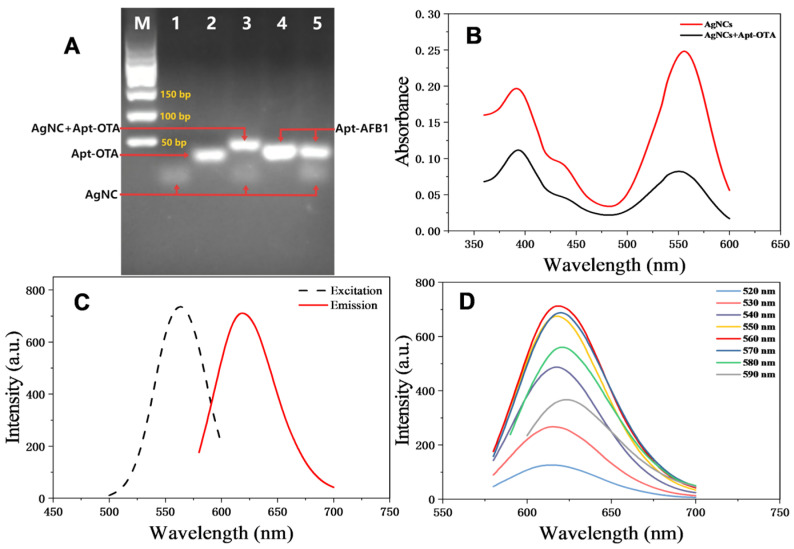
(**A**) Results of 4% agarose gel electrophoresis (lane 1: AgNCs, lane 2: Apt-OTA, lane 3: AgNCs + Apt-OTA, lane 4: aflatoxin B1 aptamer (Apt-AFB1), lane 5: AgNCs + Apt-AFB1). (**B**) Ultraviolet–visible absorption spectra of AgNCs and AgNCs + Apt-OTA. (**C**) Excitation and emission spectra and (**D**) fluorescence purity of A1-templated AgNCs.

To confirm the successful synthesis of AgNCs, we analysed free AgNCs and those conjugated with Apt-OTA (AgNCs + Apt-OTA) using TEM. The AgNCs comprised well-dispersed spherical nanoparticles with a uniform size distribution (2.71–7.73 nm, average ≈ 4.65 nm) (Figure 2), whereas larger particles (14.57–65.75 nm) were observed for AgNCs + Apt-OTA (Figure 2). The notable increase in AgNC particle size upon hybridization with Apt-OTA arises from the synergistic effects of multiple mechanisms rather than simple aggregation. First, the 38-nucleotide single-stranded Apt-OTA (Table S1) contributes to initial size expansion: its unhybridized segments project outward from AgNC surfaces as “molecular tails” to increase hydrodynamic radius, while exposed nucleobases of adjacent Apt-OTA strands form weak non-covalent interactions (hydrogen bonding, hydrophobic stacking) that drive oligomeric association of Apt-OTA-bound AgNCs. Second, the inherent negative charge of DNA-templated AgNCs (from phosphate backbones) is modulated by Apt-OTA binding: excess surface negative charge attracts buffer counterions (Na^+^, K^+^) to form an electrical double layer, reducing electrostatic repulsion between AgNCs, and the ordered hybrid duplex redistributes surface charge to further weaken repulsive forces that maintain monodispersity. Third, linear Apt-OTA acts as a cross-linker, with one segment hybridising to the template of one AgNC and another segment (via partial complementarity or non-specific ssDNA interactions) binding to adjacent AgNCs, forming large interconnected aggregates. This mechanism is corroborated by agarose gel electrophoresis (Figure 3), where a high-molecular-weight band for the AgNC-Apt-OTA complex (lane 3) confirms complex formation rather than physical adsorption, and no significant size increase with non-complementary aptamers (e.g., Apt-AFB1, lane 5) validates the specificity of the interaction. These observations confirmed the formation of the AgNCs.

To confirm the formation of AgNCs and their hybridisation with Apt-OTA, we probed five samples (lane 1: AgNCs, lane 2: Apt-OTA, lane 3: AgNCs + Apt-OTA, lane 4: Apt-AFB1, lane 5: AgNCs + Apt-AFB1) by agarose gel electrophoresis. Faint AgNC bands were visible in lanes 1, 3, and 5, and a new band corresponding to the AgNC +Apt-OTA complex was observed in lane 3 (Figure 3). By comparing lanes 3 and 5, we demonstrated the specificity of Apt-OTA binding by the AgNCs.

The UV–vis absorption spectrum of AgNCs (Figure 3) featured a typical peak of silver nanoparticles (AgNPs) at 392 nm and that of AgNCs at 556 nm. The latter peak closely matched the excitation wavelength, indicating an excellent consistency. Upon the hybridisation with Apt-OTA (Figure 3), the AgNP peak lost intensity, whereas that of AgNCs + Apt-OTA not only lost intensity but also shifted to longer wavelengths by 6 nm. These findings suggest the formation of a new complex following the incubation of AgNCs with Apt-OTA. The detection of the AgNP peak at 392 nm alongside AgNCs is attributed to trace amounts of AgNPs formed as inevitable byproducts during AgNC synthesis, rather than a deviation from the target product. The synthesis of DNA-templated AgNCs involves the reduction of Ag^+^ to Ag atoms by NaBH_4_, followed by Ag atom assembly into AgNCs (2.71–7.73 nm, Figure 2) mediated by the cytosine-rich DNA template. However, a small fraction of Ag atoms may undergo uncontrolled aggregation into larger, non-template-bound AgNPs (typically > 10 nm), which exhibit the characteristic surface plasmon resonance (SPR) peak at ~392 nm—consistent with reported AgNP UV-vis behaviour. Importantly, these trace AgNPs do not interfere with AgNC signals: (1) The AgNC peak at 556 nm is distinct from the 392 nm AgNP peak and aligns with the AgNC excitation wavelength (560 nm), ensuring no spectral overlap in fluorescence measurements; (2) TEM images (Figure 2) confirm AgNCs as the dominant product, with AgNPs present only in negligible quantities; (3) Fluorescence spectra of AgNCs (Figure 3) show stable, intense red emission (620 nm) unaffected by AgNPs, as AgNPs lack the fluorescence properties of AgNCs. This minor byproduct formation is common in DNA-templated AgNC synthesis and does not compromise the aptasensor’s performance.

Further UV-vis analysis revealed concentration-dependent trends under fixed Apt-OTA concentration: decreasing AgNC concentration reduced the intensity of both AgNC and AgNP peaks. This reduction is driven by two synergistic Apt-OTA effects: (1) Apt-OTA-AgNC interaction modifies AgNC surface electron cloud distribution, disrupting SPR and weakening absorption; (2) Apt-OTA induces AgNC aggregation, which increases particle size, enhances light scattering, reduces effective absorption area, and alters the local electromagnetic field to further impact SPR-related absorption. Even trace AgNPs show reduced peak intensity, reflecting Apt-OTA’s indirect effect on the entire nanoparticle system’s dispersion. Collectively, these effects explain Apt-OTA-induced weakening of both AgNC and AgNP peaks.

The AgNCs exhibited an excitation peak of 562 nm and an emission peak of 620 nm, with the Stokes shift therefore equalling 58 nm (Figure 3). Upon excitation within the range of 520–590 nm, the emission wavelength of the AgNCs shifted between 614 nm and 626 nm. This consistent emission range indicated the presence of a single type of AgNCs, which emitted pure red fluorescence (Figure 3).

### 3.5. Apt-OTA Parameter Optimisation

**Figure 4:**
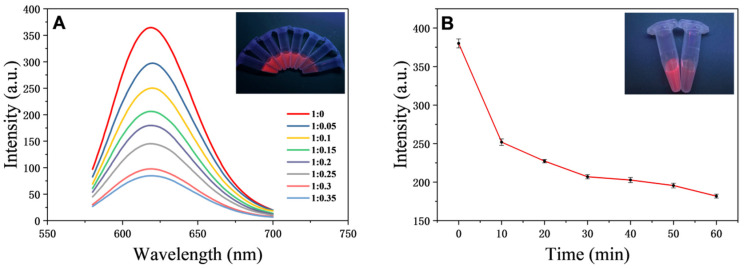
(**A**) Fluorescence spectra of AgNCs incubated with Apt-OTA aptamers at different molar ratios (Apt-OTA concentration = 0.5–3.5 μM, AgNC concentration = 10 μM. The two solutions were mixed in a 1:1 *v*/*v* ratio). (**B**) Effect of incubation time on the extent of fluorescence quenching by Apt-OTA (Apt-OTA concentration = 1.5 μM, AgNC concentration = 10 μM. The two solutions were mixed in a 1:1 *v*/*v* ratio).

Given that Apt-OTA acts as both a recognition and quenching agent for AgNCs, the concentration of the AgNCs in the presence of Apt-OTA is another critical factor influencing the OTA sensing performance. As the AgNC (A1 templated):Apt-OTA molar ratio increased (1:0.05 → 1:0.1 → 1:0.15 → 1:0.2 → 1:0.25 → 1:0.3 → 1:0.35), so did the quenching efficiency (19.58% → 31.93% → 43.59% → 51.47% → 60.13% → 73.18% → 77.17%) (Figure 4). Theoretically, the Apt-OTA concentration should be sufficiently high to ensure effective quenching yet not sufficiently high to induce non-specific background signals. As a result, a molar ratio of 1:0.15 was chosen to achieve an optimal balance between sensitivity and linear range. The hybridisation time of Apt-OTA substantially influenced the quenching efficiency and was negatively correlated with the AgNC fluorescence intensity. The fluorescence intensity at a quenching time of 40 min minimally exceeded that at 30 min, which indicated that saturation was reached after 30 min of hybridisation (Figure 4). In addition, the quenching efficiency increased by 84.5% within the 0–30 min hybridization period. Hence, the optimal incubation period was determined as 30 min.

### 3.6. Sensitivity of OTA Detection

**Figure 5:**
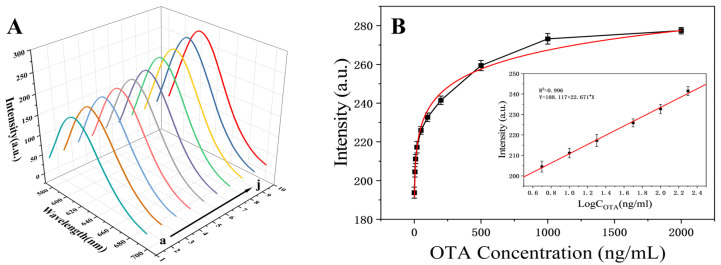
(**A**) Fluorescence spectra of the aptasensor at different OTA concentrations. (**B**) Effects of OTA concentration on the fluorescence intensity extracted from the spectra in (**A**). Inset shows the corresponding logarithmic plot. Error bars were obtained from three experiments (5–200 ng/mL).

The sensitivity of OTA detection was evaluated using OTA concentrations of 0–2000 ng/mL under the optimised conditions. The fluorescence intensity increased with the increasing OTA concentration (Figure 5) and was linearly correlated with the logarithm of the latter (Figure 5). Notably, the inset of Figure 5 further depicts the linear correlation between fluorescence intensity and the logarithm of OTA concentrations within the 5–200 ng/mL range (R^2^ = 0.996), with this low-to-mid concentration range—a subset of the full 0–2000 ng/mL data—selected for the inset: linear responses (e.g., the aptasensor’s fluorescence-concentration correlation) hold exclusively within it, it aligns with practical OTA detection focusing on trace levels in foods, and data here exhibit smaller sample preparation and instrumental errors to secure the aforementioned high linear correlation coefficient (R^2^ = 0.996) for the fitting equation. The limit of detection (LOD), i.e., the concentration corresponding to a fluorescence signal three times the standard deviation of the blank sample in the absence of OTA divided by the slope of the calibration curve (3σ/slope), was calculated as 0.38 ng/mL.

### 3.7. OTA Detection Specificity

**Figure 6:**
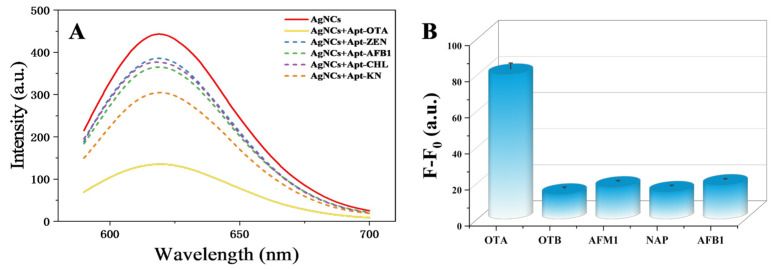
Specificity of (**A**) AgNC binding by different aptamers and (**B**) OTA detection in the presence of different mycotoxins.

The selectivity of the sensor was evaluated from two perspectives. The nature of the quenching effect exerted by Apt-OTA was evaluated using four randomly chosen aptamers, namely Apt-ZEN, Apt-AFB1, Apt-CHL, and Apt-KAN, at a AgNC:aptamer molar ratio of 1:0.15. Fluorescence quenching occurred exclusively in the case of Apt-OTA (Figure 6). Subsequently, we examined the specificity of Apt-OTA for detecting various mycotoxins (OTA, OTB, AFM1, NAP, and AFB1 standards at 100 ng/mL). Upon the addition of OTA, the fluorescence of AgNCs substantially increased compared with that observed in the presence of other mycotoxins, demonstrating the outstanding specificity of Apt-OTA detection (Figure 6).

When the AgNCs were combined with Apt-OTA (lane 3), a new bright band not observed for pure AgNCs (lane 1) and Apt-OTA (lane 2) emerged (Figure 3). In addition, the intensity of the Apt-OTA band noticeably decreased in the mixture of AgNCs and Apt-OTA. No novel bands appeared when the AgNCs were paired with Apt-AFB1 (lane 5). Thus, Apt-OTA had a distinct quenching effect on the AgNCs.

These experimental results collectively validate the excellent specificity of the AgNC-based OTA aptasensor, as it can specifically interact with Apt-OTA (but not other irrelevant aptamers) and selectively respond to OTA (but not other structurally similar mycotoxins)—with agarose gel electrophoresis further confirming this specific binding—thus ensuring reliable and accurate OTA detection in complex matrices.

### 3.8. Evaluation of OTA in Practical Samples

The developed OTA detection system was evaluated under real-life conditions using the standard addition method to demonstrate its practicality and reliability, with OTA-free red wine used as the base matrix. The wine was spiked with OTA to concentrations of 20, 60, 100, 140, and 180 ng/mL, and the samples were diluted 100-fold and analysed using the developed system. The recovery rates ranged from 97.2% to 104%, with the corresponding relative standard deviations ranging from 3.47% to 5.54% (Table 1). To assess whether the developed fluorometric aptasensor is suitable for real sample detection, this methodology was validated against a conventional technique (HPLC), and relevant experimental details can be found in the Supporting Information. Notably, the OTA concentrations measured by HPLC were also in good agreement with the spiked concentrations. Before being utilised, all OTA samples underwent filtration through a 0.4 μm filter. These findings indicate the suitability of the AgNC-based label-free aptasensor for reproducibly detecting OTA in complex matrices.

## 4. Conclusions

An unlabelled, straightforward, and highly sensitive aptasensor for OTA detection was developed using AgNCs with aptamer-controlled fluorescence, via a simplified and cost-effective DNA modification process. Key parameters (e.g., reagent dosage) were fine-tuned to optimise performance, with optimal AgNC synthesis conditions identified as PB buffer (pH 7.4), DNA:Ag^+^:NaBH_4_ molar ratio 1:10:5, and 4 °C incubation—these adjustments enabled superior specificity, stability, higher sensitivity, and a linear range covering trace OTA residues to high-concentration contamination in food samples. The sensor operates via aptamer-mediated regulation: the strong binding affinity between OTA and its aptamer (Apt-OTA) prevents Apt-OTA from quenching AgNC fluorescence, realising fluorescence activation-based detection. Apt-OTA fulfils both target recognition and signal quenching roles, eliminating the need for separate quenching agents or amplification steps; moreover, fluorescence activation mitigates false positives caused by environmental interference. Notably, most existing AgNC-based OTA sensors are limited to solid grain samples, while liquid food matrices (e.g., red wine) have long posed challenges—alcohol and polyphenols in such liquids disrupt AgNC stability and aptamer-OTA binding, leading to inaccuracies. We addressed this by validating our sensor in red wine (via comparison with gold-standard HPLC), confirming its substantial stability and specificity for OTA in complex liquids and filling the gap in rapid liquid food OTA detection. With a limit of detection (LOD) of 0.38 ng/mL and 60 min testing duration, the sensor is suitable for point-of-care applications, and this approach holds considerable potential for quantitative mycotoxin detection in food samples.

## Figures and Tables

**Table 1:** Recoveries of OTA in spiked red wine samples (n = 3).

Sample Number	Added (ng/mL)	Detected (ng/mL)		Recovery (%)		Relative Standard Deviation (%)	
		Present Method	HPLC	Present Method	HPLC	Present Method	HPLC
1	20	20.8	19.5	104	97.5	4.17	4.27
2	60	59.7	61.4	99.5	102.3	3.47	3.78
3	100	97.2	102.1	97.2	102.1	5.54	5.06
4	140	141.2	139.6	100.9	99.7	4.48	4.47
5	180	183.9	181.2	102.2	100.7	5.27	5.53

## Data Availability

The original contributions presented in the study are included in the article/Supplementary Materials, further inquiries can be directed to the corresponding author.

## Supplementary Materials

The following supporting information can be downloaded at: https://www.mdpi.com/article/10.3390/foods14183271/s1, Table S1: The oligonucleotide sequences used in the template sequence selection. Figure S1: The complementary sites between the Apt-OTA and DNA templates (A1~A5). Figure S2: The optimization of the AgNCs synthesis conditions. Table S2: Comparison of different methods for determination of OTA. References [38,41,42,43,44,45,46,47] are cited in Table S2.

## References

[1] Bryła M., Ksieniewicz-Woźniak E., Stępniewska S., Modrzewska M., Waśkiewicz A., Szymczyk K., Szafrańska A. (2021). Transformation of ochratoxin A during bread-making processes. Food Control.

[2] Gu K., Ryu D., Lee H.J. (2021). Ochratoxin A and its reaction products affected by sugars during heat processing. Food Chem..

[3] Wang L., Hua X., Shi J., Jing N., Ji T., Lv B., Liu L., Chen Y. (2022). Ochratoxin A: Occurrence and recent advances in detoxification. Toxicon.

[4] Kumar P., Mahato D.K., Sharma B., Borah R., Haque S., Mahmud M.M.C., Shah A.K., Rawal D., Bora H., Bui S. (2020). Ochratoxins in food and feed: Occurrence and its impact on human health and management strategies. Toxicon.

[5] Tao Y., Xie S., Xu F., Liu A., Wang Y., Chen D., Pan Y., Huang L., Peng D., Wang X. (2018). Ochratoxin A: Toxicity, oxidative stress and metabolism. Food Chem. Toxicol..

[6] Klingelhöfer D., Braun M., Schöffel N., Oremek G.M., Brüggmann D., Groneberg D.A. (2020). Ochratoxin–Characteristics, influences and challenges of global research. Food Control.

[7] Wang L., Wang Q., Wang S., Cai R., Yuan Y., Yue T., Wang Z. (2022). Bio-control on the contamination of Ochratoxin A in food: Current research and future prospects. Curr. Res. Food Sci..

[8] Yang Q., Dhanasekaran S., Ngea G.L.N., Tian S., Li B., Zhang H. (2022). Unveiling ochratoxin a controlling and biodetoxification molecular mechanisms: Opportunities to secure foodstuffs from OTA contamination. Food Chem. Toxicol..

[9] Wei W., Liu C., Ke P., Chen X., Zhou T., Xu J., Zhou Y. (2021). Toxicological and physiological effects of successive exposure to ochratoxin A at food regulatory limits. Food Chem. Toxicol..

[10] Campone L., Piccinelli A.L., Celano R., Pagano I., Russo M., Rastrelli L. (2018). Rapid and automated on-line solid phase extraction HPLC–MS/MS with peak focusing for the determination of ochratoxin A in wine samples. Food Chem..

[11] Njumbe Ediage E., Van Poucke C., De Saeger S. (2015). A multi-analyte LC–MS/MS method for the analysis of 23 mycotoxins in different sorghum varieties: The forgotten sample matrix. Food Chem..

[12] Yang X.-S., Zhao J., Ma T.-T., Li Z.-Y., Wang L.-L., Ji S.-L., Sun M.-Y., Liu Y.-S., Hu Z.-H., Liu Q.-W. (2023). Magnetic covalent organic framework for effective solid-phase extraction and HPLC determination of ochratoxin A in food. Lwt.

[13] Ouyang M., Liu T., Yuan X., Xie C., Luo K., Zhou L. (2025). Nanomaterials-based aptasensors for rapid detection and early warning of key food contaminants: A review. Food Chem..

[14] Xavier M., Parente I.A., Rodrigues P.M., Cerqueira M.A., Pastrana L., Gonçalves C. (2021). Safety and fate of nanomaterials in food: The role of in vitro tests. Trends Food Sci. Technol..

[15] Eguílaz M., Gutierrez F., González-Domínguez J.M., Martínez M.T., Rivas G. (2016). Single-walled carbon nanotubes covalently functionalized with polytyrosine: A new material for the development of NADH-based biosensors. Biosens. Bioelectron..

[16] Guo Z., Tian J., Cui C., Wang Y., Yang H., Yuan M., Yu H. (2021). A label-free aptasensor for turn-on fluorescent detection of ochratoxin a based on SYBR gold and single walled carbon nanohorns. Food Control.

[17] Wu G., Qiu H., Liu X., Luo P., Wu Y., Shen Y. (2023). Nanomaterials-based fluorescent assays for pathogenic bacteria in food-related matrices. Trends Food Sci. Technol..

[18] Kheirkhahnia S., Sadeghi S. (2025). Copper metallic nanoclusters encapsulated within zinc-based metal organic framework for optical sensing of ciprofloxacin in aqueous solutions. J. Photochem. Photobiol. A: Chem..

[19] Xu J., Bi Y., Zhao H., Shi L., Zhang N., Xin X. (2025). In situ synthesis of well-dispersed silver nanoparticles from silver nanoclusters hydrogel for catalytic reduction of 4-Nitrophenol. Appl. Surf. Sci..

[20] Xu J., Zhu X., Zhou X., Khusbu F.Y., Ma C. (2020). Recent advances in the bioanalytical and biomedical applications of DNA-templated silver nanoclusters. TrAC Trends Anal. Chem..

[21] Yang M., Zhu L., Yang W., Xu W. (2023). Nucleic acid-templated silver nanoclusters: A review of structures, properties, and biosensing applications. Coord. Chem. Rev..

[22] Zhou B., Khan I.M., Ding X., Niazi S., Zhang Y., Wang Z. (2024). Fluorescent DNA-Silver nanoclusters in food safety detection: From synthesis to application. Talanta.

[23] Petty J.T., Zheng J., Hud N.V., Dickson R.M. (2004). DNA-Templated Ag Nanocluster Formation. J. Am. Chem. Soc..

[24] Sharma J., Rocha R.C., Phipps M.L., Yeh H.-C., Balatsky K.A., Vu D.M., Shreve A.P., Werner J.H., Martinez J.S. (2012). A DNA-templated fluorescent silver nanocluster with enhanced stability. Nanoscale.

[25] Guo Y., Pan X., Zhang W., Hu Z., Wong K.-W., He Z., Li H.-W. (2020). Label-free probes using DNA-templated silver nanoclusters as versatile reporters. Biosens. Bioelectron..

[26] Yang S.W., Vosch T. (2011). Rapid Detection of MicroRNA by a Silver Nanocluster DNA Probe. Anal. Chem..

[27] Liu Y., Guan B., Xu Z., Wu Y., Wang Y., Ning G. (2023). A fluorescent assay for sensitive detection of kanamycin by split aptamers and DNA-based copper/silver nanoclusters. Spectrochim. Acta Part A: Mol. Biomol. Spectrosc..

[28] Xu D., Shan L., Guo B., Wang J., Huang Q., Wang S., Li F., Wu S., Wang W., Chen J. (2025). A fluorescent aptasensor for kanamycin detection in milk, seafood and water samples using DNA-AgNCs and exonuclease I-assisted recycling amplification strategy. Food Chem..

[29] Fan P., Li Q., Zhang Z., Ni S., Jiang P., Sun S., Li L. (2025). A novel and universal dual-channel signal amplification aptasensing platform for ultrasensitive and rapid detection of cardiac biomarkers based on the mutual regulation of bimetallic organic framework and silver nanoclusters. Talanta.

[30] Yin Y., Han L., Liu J., Xu Y., Tian Y., Mou Y., Chen D., Sun X., Guo Y., Li F. (2025). Dual-color AgNCs-enabled fluorescent aptasensor for the simultaneous detection of KAN and AFB1. Food Chem..

[31] Wu Y., Feng Y., Mei X., Zhu Y., Wu K., Deng A., Li J. (2025). A visualizable-quantitative SERS-LFIA platform for ultrasensitive detection of ochratoxin a using size-tunable Au@Ag core-shell nanocubes as the substrate. Microchem. J..

[32] Cruz-Aguado J.A., Penner G. (2008). Determination of Ochratoxin A with a DNA Aptamer. J. Agric. Food Chem..

[33] Zhou W., Zhu J., Fan D., Teng Y., Zhu X., Dong S. (2017). A Multicolor Chameleon DNA-templated Silver Nanocluster and Its Application for Ratiometric Fluorescence Target Detection with Exponential Signal Response. Adv. Funct. Mater..

[34] Kondo J., Tada Y., Dairaku T., Saneyoshi H., Okamoto I., Tanaka Y., Ono A. (2015). High-Resolution Crystal Structure of a Silver(I)–RNA Hybrid Duplex Containing Watson-Crick-like C-Silver(I)-C Metallo-Base Pairs. Angew. Chem. Int. Ed..

[35] New S.Y., Lee S.T., Su X.D. (2016). DNA-templated silver nanoclusters: Structural correlation and fluorescence modulation. Nanoscale.

[36] Li J., Wang W., Sun D., Chen J., Zhang P.-H., Zhang J.-R., Min Q., Zhu J.-J. (2013). Aptamer-functionalized silver nanoclusters-mediated cell type-specific siRNA delivery and tracking. Chem. Sci..

[37] Li J., Zhong X., Cheng F., Zhang J.-R., Jiang L.-P., Zhu J.-J. (2012). One-Pot Synthesis of Aptamer-Functionalized Silver Nanoclusters for Cell-Type-Specific Imaging. Anal. Chem..

[38] Li R., Zhu L., Yang M., Liu A., Xu W., He P. (2024). Silver nanocluster-based aptasensor for the label-free and enzyme-free detection of ochratoxin A. Food Chem..

[39] Lin Y.-H., Tseng W.-L. (2009). Highly sensitive and selective detection of silver ions and silver nanoparticles in aqueous solution using an oligonucleotide-based fluorogenic probe. Chem. Commun..

[40] Ono A., Cao S., Togashi H., Tashiro M., Fujimoto T., Machinami T., Oda S., Miyake Y., Okamoto I., Tanaka Y. (2008). Specific interactions between silver(i) ions and cytosine–cytosine pairs in DNA duplexes. Chem. Commun..

[41] Xu L., Qu W., Zhu Z., Hao X., Yang Q., Liu L., Gong Z., Li P. (2025). Tetrahedral DNA scaffold-based label-free electrochemical aptasensor for the detection of ochratoxin A. Lwt.

[42] Briones N., Gómez H., Henríquez R., Rojas V., Villarroel M. (2025). Photoelectrochemical aptasensor for detection of Ochratoxin A in synthetic red wine samples. J. Photochem. Photobiol. A: Chem..

[43] Xu M., Li Y., Hu X., Zhang S., Liu Y. (2025). Smartphone-assisted antifouling colorimetric aptasensor for ultrasensitive ochratoxin A detection in coffee. Microchem. J..

[44] Li Y.-L., Chen Y.-Y., Xie F.-T., Li Q.-X., Yang T., Yang Y.-H., Hu R. (2025). Smartphone-based dual-mode aptasensor with bifunctional metal-organic frameworks as signal probes for ochratoxin A detection. Food Chem..

[45] Chen J., Zhang L., Yu R. (2024). Nucleic acid aptamer based thermally oxidized porous silicon/zinc oxide microarray chip for detection of ochratoxin A in cereals. Food Chem..

[46] Zhao Y., Niu M., Wei D., Wang B., Guan T., Lv L., Guo Z. (2025). Exonuclease III-mediated label-free fluorometric aptasensor for ochratoxin A detection in food samples based on aggregation-induced emission probe. J. Food Compos. Anal..

[47] Xu H., Xiao C., Zhao F., Suo Z., Liu Y., Wei M., Jin B. (2025). Ratiometric fluorescent aptasensor based on DNA-gated Fe3O4@Uio-66-NH2 and Exo I-assisted signal amplification. Analytica Chimica Acta.

