# Supplementary material for: Silver Nanocluster–Based Label-Free Aptasensor for the Turn-On Fluorescent Detection of Ochratoxin A
Source: Foods. 2025 Sep 20;14(18):3271. doi: 10.3390/foods14183271 (PMC12469903; doi:10.3390/foods14183271)
Supplement: Supplementary file 1 [file foods-14-03271-s001.zip › foods-3849744-supplementary.pdf]

Supporting information

Silver Nanocluster–Based Label-Free Aptasensor for the Turn-On Fluorescent Detection of Ochratoxin A

Jinyan Nan, Chengbi Cui and Zhijun Guo *

College of Agriculture, Yanbian university, Yanji 133002, China; zjguo@ybu.edu.cn

Email: guozhijunvip@163.com

Fax: (+86)-433-2435549; Tel: (+86)-433-2435549

Table. S1. The oligonucleotide sequences used in the template sequence selection.

No.	Sequence (5′-3′)
S1	**CCC CCT TAA TCC CCC** TAT AAT AAA TTT
S2	**CCC CCT TAA TCC CCC** TAT AAT AAA TTT TAA ATA TTA
S3	**CCC CCT TAA TCC CCC** TGA GGC GAT GTT
S4	**CCC CCT TAA TCC CCC** TCA CCG CAT CTT
S5	**CCC CCT TAA TCC CCC** AGT CAC CCC AAC
S6	**CCC CCT TAA TCC CCC** TGA GGC GAT G
A1	CCC CCT TAA TCC CCC ACG CCA CCC ACA CC
A2	CCC CCT TAA TCC CCC ACG CCA CCC ACA CCC
A3	CCC CCT TAA TCC CCC TCC CTT TAC GCC AC
A4	CCC CCT TAA TCC CCC TCC GAT GCT CCC TT
A5	CCC CCT TAA TCC CCC TGT CCG ATG CTC
Apt-OTA	GAT CGG GTG TGG GTG GCG TAA AGG GAG CAT CGG ACA

Note: The underlined sequences are taken from the complementary chain of the Apt-OTA (C-OTA); the bold sequence is the nucleation sequence of the AgNCs.

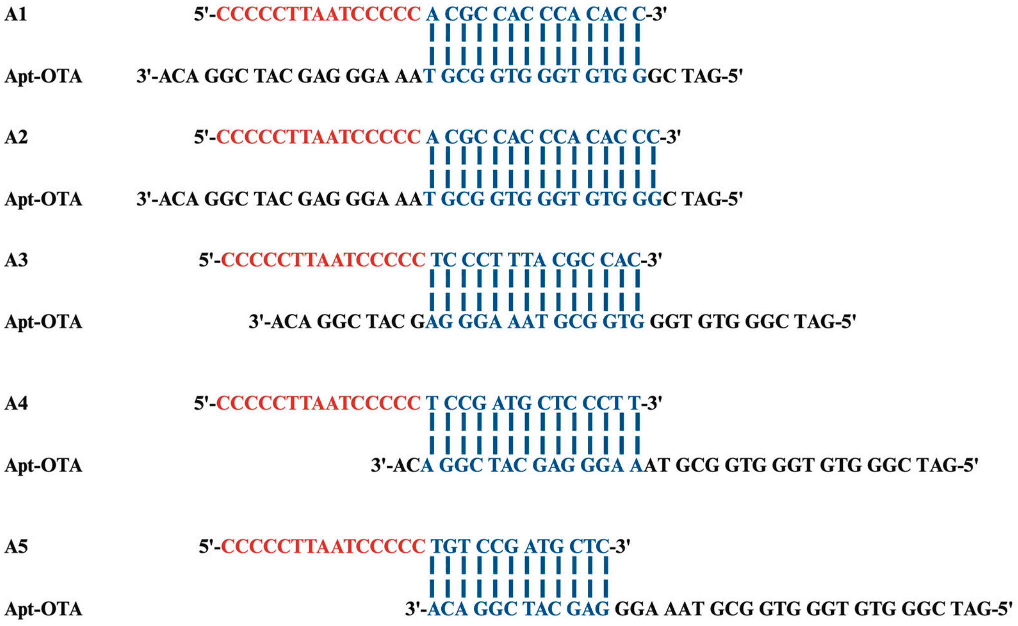

Fig.S1. The complementary sites between the Apt-OTA and DNA templates (A1~A5). (**Note:** A1 refers to the binding of complementary sites between AgNCs and the

Apt-OTA used in this experiment.)

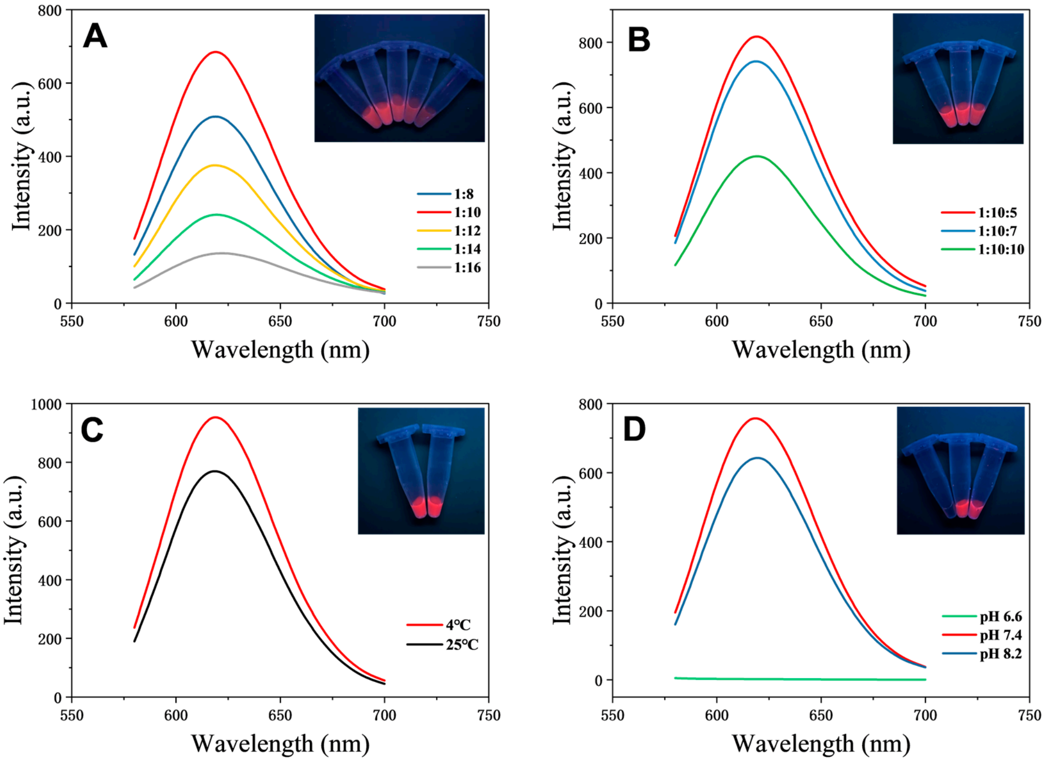

Fig.S2. The optimization of the AgNCs synthesis conditions. (A) The DNA template to Ag^{+} ratio. (B) The DNA template to Ag^{+} to $NaBH_4$ ratio. (C) The synthesis temperature. (D) The pH value of the PB buffer (AgNCs concentration: 10 μM).

Pretreatment of Red Wine Samples and OTA Spiking Procedure

1. Pretreatment of Red Wine Samples

The pretreatment process was optimized to reduce matrix interference (e.g., polyphenols, ethanol, and residual sugars in wine) while preserving OTA stability:

- Homogenization and pH Adjustment: 10.0 mL of well-homogenized red wine was transferred to a 50 mL polypropylene centrifuge tube and vortexed for 2 min to ensure uniform mixing. The pH of the wine sample was adjusted to 7.4 ± 0.1 using 0.1 M phosphate buffer (PB, pH 7.4) under continuous vortexing—this pH condition was consistent with the optimal environment for Apt-OTA/AgNCs interaction (Section 2.3) and avoided protonation/deprotonation of OTA (a weak acid) that might affect its binding to Apt-OTA.
- Impurity Removal: The pH-adjusted wine sample was centrifuged at 3000 rpm for 5 min at room temperature to precipitate insoluble impurities (e.g., grape pulp residues and colloidal particles). The supernatant was carefully transferred to a new 50 mL centrifuge tube, and the sediment was discarded.
- Dilution: The collected supernatant was diluted 100-fold with Tris buffer (10 mM Tris, 120 mM NaCl, 20 mM $CaCl_2$, 5 mM KCl, pH 7.4) to reduce the inhibitory effect of high ethanol content (≈12–14% v/v in wine) on AgNCs fluorescence and prevent non-specific binding between wine polyphenols and Apt-OTA.

2. OTA Spiking Procedure

To evaluate the accuracy and recovery of the aptasensor, OTA-spiked wine samples were prepared at five concentration levels (20, 60, 100, 140, and 180 ng/mL, based on the undiluted wine matrix):

- OTA Standard Preparation: OTA stock solution (1 mg/mL in methanol) was serially diluted with Tris buffer to obtain OTA working solutions of 2, 6, 10, 14, and 18 μg/mL.
- Spiking and Mixing: 100 μL of each OTA working solution was added to 9.9 mL of the pretreated wine supernatant (before 100-fold dilution) in a 15 mL centrifuge tube. The mixture was vortexed vigorously for 3 min to ensure homogeneous distribution of OTA, resulting in final spiked concentrations of 20, 60, 100, 140, and 180 ng/mL in the undiluted wine.
- Uniform Dilution: The spiked wine samples were then subjected to the same 100-fold dilution step as the blank wine samples (using Tris buffer) to maintain consistency in the detection system.

After pretreatment and spiking, all samples were analyzed following the fluorescent detection protocol described in Section 2.4: 94 μL of the diluted spiked/blank wine sample was mixed with 5.5 μL of 20 μM Apt-OTA, incubated at room temperature for 30 min, then combined with 75 μL of 10.7 μM AgNCs solution (1:1 v/v) for another 30 min incubation. Fluorescence intensity was measured at 560 nm excitation, with each concentration tested in triplicate.

HPLC-Fluorescence Detector Conditions

HPLC analysis was performed on an Agilent 1260 HPLC system equipped with an Agilent SB-C18 column (250 mm × 4.6 mm, 5 μm), and the column temperature was maintained at 30 °C. The mobile phase consisted of solvent A (CH_3COOH:H_2O, 1:50, v/v) and solvent B (acetonitrile), with elution performed using the following gradient: 12% B at 0 min, 20% B at 10 min, 30% B at 12 min, and 50% B at 19 min. The sample injection volume was 10 μL, and the flow rate was 1 mL/min. Fluorescence detection (FLD) was conducted using an Agilent 1260 G1321C Fluorescence Detector, with an excitation wavelength (λ_{ex}) of 333 nm and an emission wavelength (λ_{em}) of 440 nm.

Table. S2. Comparison of different methods for determination of OTA.

No.	Methods	Materials	LOD	Reference
1	Electrochemical aptasensor	Tetrahedral DNA nanostructure (TDN)	0.68 ng/L	(Xu, Qu et al. 2025)
2	Photoelectrochemical aptasensor	ZnO-NRs/CdS/Au-NPs	0.5 μg/L	(Briones, Gómez et al. 2025)
3	Colorimetric aptasensor	Au/CeO_2/rGO nanohybrid	27.3 pg/L	(Xu, Li et al. 2025)
4	A colorimetric/electrochemical/smartphone dual-mode aptasensor	Fe-MIL-88	Electrochemical mode: 0.22 fg/mL; Colorimetric mode:0.25 fg/mL; RGB mode: 0.4 fg/mL	(Li, Chen et al. 2025)
5	Microarray chip	Thermally oxidized porous silicon/zinc oxide ($PSiO_2$/ZnO) composites	0.0196 ng/kg	(Chen, Zhang and Yu 2024)
6	Fluorescence aptasensor	Exonuclease III (Exo III)	1.37 ng/mL	(Zhao, Niu et al. 2025)
7	Fluorescence aptasensor	DNA-gated Fe3O4@Uio-66-NH2 and Nucleic acid exonuclease I	0.308 ng/mL	(Xu, Xiao et al. 2025)
8	Fluorescence aptasensor	AgNC	1.3 nM（0.524 ng/mL）	(Li, Zhu et al. 2024)
9	Fluorescence aptasensor	AgNC	0.38 ng/mL	This work
